# Coordinating a growing data enterprise: strategies for facilitating multiple research-practice relationships for the public good.

**DOI:** 10.23889/ijpds.v9i5.2841

**Published:** 2024-09-18

**Authors:** Carl Frederick

**Affiliations:** 1 Office for National Statistics

It takes time, energy, and goodwill to start a data partnership. The corollary to this truism is that maintaining and expanding such partnerships will demand as much, if not more, of these resources. Learn about the strategies we are using to expand our ability to produce rigorous, policy-relevant evidence to combat poverty and inequality while staying committed to addressing the needs of our long-term partners.

Developed by the University of Wisconsin-Madison’s Institute for Research on Poverty (IRP), the Wisconsin Administrative Data Core (WADC) evolved from a series of large-scale evaluation projects conducted for the state starting in the 1980s. This data enterprise, built by linking administrative data from multiple agencies, allows for cross-program analyses that are otherwise impossible.

Increased turnover since the pandemic coupled with more formalized data governance structures has highlighted the importance of maintaining good relationships and providing value back to partners who devote scarce bandwidth to sharing data. At the same time, we are adding new agency partners and working to increase usage of the WADC. These changes multiply the complexity involved in coordinating the data enterprise. We are adapting to this new world by focusing on relationships: creating formal structures that deepen the network of direct relationships among agency staff and researchers, taking advantage of increased turnover by hiring staff who have worked in state agencies, and seeking agency partners who want to be active partners versus data providers. More solid relationships will yield more policy-relevant research that agencies can use to better serve the state.

